# Discovery and informing research participants of incidental findings detected in brain magnetic resonance imaging studies: Review and multi‐institutional study

**DOI:** 10.1002/brb3.676

**Published:** 2017-03-29

**Authors:** Kyoko Takashima, Yoshiyuki Takimoto, Eisuke Nakazawa, Yoshinori Hayashi, Atsushi Tsuchiya, Misao Fujita, Akira Akabayashi

**Affiliations:** ^1^ Department of Public PolicyInstitute of Medical Sciencethe University of TokyoTokyoJapan; ^2^ Department of Biomedical EthicsGraduate School of Medicinethe University of TokyoTokyoJapan; ^3^ Department of PhilosophyCollege of LettersRitsumeikan UniversityKyotoJapan; ^4^ Graduate School of Integrated Arts and SciencesTokushima UniversityTokushimaJapan; ^5^ Uehiro Research Division for iPS Cell EthicsCenter for iPS Cell Research and Application (CiRA)Kyoto UniversityKyotoJapan

**Keywords:** incidental findings, magnetic resonance imaging study, quantitative study, research ethics, review

## Abstract

**Background:**

Brain imaging studies using magnetic resonance imaging (MRI) sometimes reveal incidental findings (IFs) that might be relevant to some of the health issues in research participants. Although professional communities have discussed how to manage these IFs, there is no global consensus on the concrete handling procedures including how to inform participants of IFs.

**Methods:**

First, this study reviewed previous studies for the number of IFs discovered in brain imaging studies using MEDLINE. Second, a multi‐institutional study determined the number of IF discoveries and evaluated the method of informing participants at multiple institutions, which participated in a national brain science project in Japan.

**Results:**

Both the review and multi‐institutional study showed that IFs with a high urgency level were discovered in 0–2.0% of participants, including healthy volunteers, and that the rate of IF discovery in general was higher in studies conducted in elderly population. Moreover, multi‐institutional study suggested the criteria used to judge whether or not to inform participants of IFs may differ by institution.

**Conclusions:**

Our results suggest that in order to ensure informing the participants of high urgency IFs, physicians who are capable of interpreting brain images clinically should review all brain images, and the establishment of a support system is required for brain imaging studies at nonmedical institutions. Since the method of informing participants of IFs might affect their understanding and acceptance of IFs, which are related to managing risks of false “clean bill of health” or psychological impacts of informing IFs, further research focusing on communication of IFs is needed.

## 1 Introduction

Brain science studies have recently achieved great advances with the use of brain imaging technologies such as magnetic resonance imaging (MRI). Among these technologies, MRI has been widely used in studies involving healthy volunteers because it allows for noninvasive observation and measurement of brain activities. Moreover, the use of functional MRI (fMRI) is not limited to medical studies (Illes & Raffin, 2002; Rosen & Savoy, 2012). In fact, it has also expanded to nonmedical brain science studies with psychological, sociological, or economic relevance (Illes, 2003; Illes & Kirschen, 2003; Illes et al., 2008). This allowed for more opportunities for acquiring brain images in studies (Federico, Lombera, & Illes, 2011; Illes, Kirschen, & Gabrieli, 2003; Wardlaw et al., 2015). With this tendency, the handling of “incidental findings” (IFs) has become an issue. An IF is defined as “a finding concerning an individual research participant that has potential health or reproductive importance and is discovered in the course of conducting research but is beyond the aims of the study” (Wolf, Lawrenz, et al., 2008). Handling of IFs is being increasingly discussed in the field of research ethics. In December 2013, the Presidential Commission for the Study of Bioethical Issues released a report on IFs entitled “Anticipate and Communicate: Ethical Handling of Incidental and Secondary Findings in the Clinical, Research, and Direct‐to‐Consumer Contexts” (Presidential Commission for the Study of Bioethical Issues, 2013). This report specifically addresses genetic sequencing, biological specimens, and imaging.

Since the 2000s, many experts of brain imaging studies have discussed the significance of IFs (Grossman & Bernat, 2004; Illes, 2006; Illes, Desmond, Huang, Raffin, & Atlas, 2002; Illes, Rosen, et al., 2004; Illes et al., 2006; Wolf, 2008; Wolf, Lawrenz, et al., 2008). Some of the IFs that are encountered in brain imaging studies are life‐threatening and require urgent action (Hilgenberg, 2006; Underwood, 2012). Thus, the greatest benefit of discovering IFs and providing the information to participants is that it can lead to an early detection of serious diseases (Borgelt, Anderson, & Illes, 2013; Hilgenberg, 2006; Illes et al., 2002, 2006; Underwood, 2012). On the other hand, researchers are not necessarily capable of clinically evaluating images, and the imaging methods and performance of the equipment employed in studies might not be sufficient for clinical evaluation, even though it is adequate for the purpose of the study (Booth, Waldman, Wardlaw, Taylor, & Jackson, 2012; Cramer et al., 2011; Grossman & Bernat, 2004; Illes et al., 2002; Illes, Kirschen, et al., 2004; Mamourian, 2004; Wolf, Paradise, & Caga‐anan, 2008). Thus, the risks of offering information about IFs have been reported, such as the possibility of causing fear in participants, posing time, physical, and financial burden on participants for detailed examinations (Anonymous, 2005; Grossman & Bernat, 2004; Illes et al., 2006; Kumra, Ashtari, Anderson, Cervellione, & Kan, 2006; Warlow, 2011), possibility of false‐negative and false‐positive results (Illes et al., 2006; Kumra et al., 2006; Royal & Peterson, 2008), existence of a “therapeutic misconception” (Kirschen, Jaworska, & Illes, 2006; Meltzer, 2006; Miller, Mello, & Joffe, 2008; Parker, 2008; Shaw, Senior, Peel, Cooke, & Donnelly, 2008), and issues related to insurability (Apold & Downie, 2011; Check, 2005). Thus far, experts have reached a consensus that researchers are obliged to respond to IFs in some way (Wardlaw et al., 2015; Wolf, Lawrenz, et al., 2008) and proposed some models for handling IFs (Illes et al., 2008; Wolf, Lawrenz, et al., 2008; Cramer et al., 2011; NINDS (National Institute of Neurological Disorders and Stroke), 2005; Shoemaker et al., 2016). However, there is no global consensus on the concrete handling procedures (Borgelt et al., 2013; Underwood, 2012; Wardlaw et al., 2015).

One reason that the discussion has stalled despite accumulated theoretical considerations lies in the lack of empirical research (Presidential Commission for the Study of Bioethical Issues, 2013; Royal & Peterson, 2008). Shoemaker et al. (2011, 2016) established a system in which all images taken for research purposes are reviewed and evaluated by a neuroradiologist and the results are offered to all participants, and they also examined its feasibility. Such a review system is ideal for reducing the possibility of false‐positive and false‐negative interpretations. However, as mentioned above, brain imaging studies are not conducted only by medical institutions. Therefore, due to the limited access to neuroradiologists or research budget, there could be cases where such a system cannot be established, or even the research activity itself might not be pursued if such system is mandated. Moreover, there has been little research that compared the number of IFs discovered at different institutions or examined the feasibility of employing a standardized IF handling procedure, despite the fact that many studies are conducted at multiple institutions as large‐scale collaborative projects.

Given this situation, we set two objectives for this study; first, to review already published empirical studies for the number of IFs discovered during brain MRI and analyze their characteristics, and second, to find out the number of IFs, and the status and method of informing participants of their IFs among multiple institutions. In a review, while referring to a systematic‐review of IFs in MRI by Morris et al. (2009), we focus on only the research setting and cover the follow‐up study data after IF finding.

## 2 Methods

### 2.1 Review of IF reports in previous studies

We systematically searched the literature on empirical studies that reported the number of IFs discovered in brain MRI studies using MEDLINE via PubMed. The search strategy used the following keywords: (“Neuroimaging”[Mesh] OR (“Magnetic Resonance Imaging”[Mesh] AND brain)) AND “Incidental Findings”[Mesh] AND (English[lang] AND bioethics[sb]) (searched on May 31, 2016). From the search results, articles other than brain imaging studies were excluded based on the title and abstract. Then, we read the body text of all remaining articles to exclude argument‐based articles and studies other than those on the number of discovered IFs. During this process, we included appropriate literature from references of the articles that were identified in the search.

### 2.2 Comparative study of the number of IFs discovered at multiple institutions (multi‐institutional study)

#### 2.2.1 Used data

The data used and analyzed in this study were from a survey on the conduct of brain imaging studies and discoveries of IFs (Takashima, Tashiro, Tsuchiya, Fujita, & Takimoto, 2013) that was conducted by the Bioethics Working Group of the “Strategic Research Program for Brain Sciences (SRPBS)” (Ministry of Education, Culture, Sports, Science and Technology, Japan), which is a nation‐wide brain science research project started in 2008 by the Ministry of Education, Culture, Sports, Science and Technology, Japan. Under the SRPBS, numerous studies have been conducted, ranging from basic research, including development of model animals, applied research, such as the development of brain‐machine interface (BMI), elucidation of psychiatric disorders, and development of diagnostic methods and biomarker candidates. As part of this research project, brain imaging studies have been conducted using human subjects. The Bioethics Working Group comprises principal investigators (PIs) in the SRPBS and bioethics experts and has played an important role in facilitating the discussion of ethical and social issues encountered in promoting this project.

The SRPBS survey was conducted by sending e‐mails twice, in October 2012 (the first survey) and March 2013 (the second survey), to PIs at a total of 92 institutions where research under the SRPBS is conducted. PIs were asked whether or not brain imaging studies were conducted and whether IFs were discovered, respectively, in the past 6 months. Fifty‐three of the 92 institutions responded (response rate, 57.6%), and 16 institutions answered that they conducted brain imaging studies in human participants, of which 14 institutions used MRI. We used the data of these 14 institutions (including two institutions responded to only one of the e‐mails) obtained on images taken within a year from April 2012 to March 2013 from the two surveys.

The data used in this study were anonymized so that the personal information of participants could not be linked to the individuals. In addition, since our analysis involved secondary use of data, a review by the ethical committee was deemed unnecessary.

#### 2.2.2 Policy for handling of IFs under the SRPBS

In response to the recommendation regarding the handling procedure of IFs based on theoretical and investigative studies (Fujita et al., 2014), all brain imaging studies conducted under the SRPBS followed the following policy in a standardized manner:

With respect to all brain images taken during a neuroscience research study conducted by the SRPBS, it is desirable that a licensed physician performs a screening test to appropriately examine if a clear abnormality exists. For the time being, we will treat every abnormality this way on a trial basis to help shed light on unforeseen issues. Re‐examination of this method after a year is desirable (Fujita et al., 2014; Hayashi, Fujita, Takashima, Tashiro, & Akabayashi, 2012)

In this policy, a reviewing physician was defined as a physician who reviews brain images in his/her daily work and is capable of interpreting them clinically. In carrying out the above policy, researchers were asked to follow four items during the ethical review and when explaining the study to participants in the process of obtaining informed consents (ICs): description in the study protocol, explanation of handling policy of IFs in the study to participants, development of a system to evaluate the images, and informing research participants of findings (Table 1). The criteria and method of informing research participants of IFs were established and performed at individual institutions, taking their respective circumstances into consideration.

**Table 1:** Consideration in adopting the incidental finding (IF) handling policy of SRPBS

1. Description in the study protocol Researchers should include the content of this policy as the handling procedure for IFs, in the study protocol of newly conducted brain imaging studies. In addition, regarding the studies that have already been approved by the research ethics committee, if imaging is planned in the future, it is desirable that researchers promptly apply to the research ethics committee for a change that reflects the content of this policy.
2. Explanation of the study to participantsResearchers should explain the following in writing prior to the conduct of the study to obtain consent for participation in the study. Brain images are taken for no purpose other than research, and not for the purpose of clinical diagnosis. Images taken are not necessarily appropriate for clinical diagnosis. All images are subjected to a general evaluation by physicians. If any findings that need further examination are incidentally discovered during the course of the aforementioned evaluation, researchers will inform the participants. This research does not assume the cost or other liabilities incurred in new visits to medical institutions for a detailed examination of the discovered findings.
3. Development of a system to evaluate the images A system of image evaluation by physicians should be developed. All brain images should be evaluated by physicians (radiologists and other physicians who are capable of interpreting brain images clinically) at a maximum interval of half a year. When clear abnormalities are found, the research participant in question should be advised to seek for further examination at medical institutions. If it is difficult for research institutions to develop a system for the evaluation of images by such physicians due to a lack of appropriate medical facilities in the same department or other reasons, physicians can be sent from the SRPBS to the research institutions. When a physician sent from the SRPBS finds IFs, the participant in question should receive a letter advising further detailed investigation at medical institutions that was mailed or handed over from the research institution.
4. Informing research participants of findings Criteria and procedures for disclosing IFs to research participants should be decided based on the circumstances of individual research institutions. Fact‐finding investigations should be conducted for future discussion on the handling of IFs. Whether or not to accept the participants’ choice to remain uninformed of IFs should be left to the discretion of individual research institutions.

#### 2.2.3 Items regarding IFs

From the above survey results, we used the following data: the number of research participants whose brain images were taken, the number of participants in whom IFs were discovered, basic attributes of IFs, types of equipment used for taking images, types of images, types of individual findings (suspected disease names), judgment of the urgency level of findings (one of the four levels of Immediate, Urgent, Routine, or No referral (Illes, Rosen, et al., 2004; Shoemaker et al., 2011; Katzman, Dagher, & Patronas, 1999; Seki, Uchiyama, Fukushi, Sakura, & Tatsuya, 2010; Kim, Illes, Kaplan, Reiss, & Atlas, 2002)), whether or not research participants were informed of the findings, and the informing method used when informed.

### 2.3 Statistical analysis

We investigated the associations between detection rates and the following variables: participants’ age, sex, and whether a patient or a healthy volunteer. Since we could not obtain the information on age of all participants whose images were taken, we classified the institutions into two groups based on the subject of their research projects (Takashima et al., 2013). Each institution conducted one research project under SRPBS, and they were assigned to either of the groups, namely, the elderly research group or nonelderly research group. The former group comprised institutions where research participants were limited to middle‐aged or elderly individuals (for research on depression in middle‐aged individuals and on dementia). Next, chi‐square tests were performed using the total number of participants for whom images were taken and the total number of IFs discovered for each group. In addition, t‐tests were performed using those numbers for each institution.

To compare sexes and patients versus healthy volunteers, only the second survey results were used. Chi‐square tests were performed using the total number of participants whose images were taken and discovered IFs of relevant institutions. In addition, Cochran‐Mantel‐Haenszel (CMH) tests were performed using those numbers for each institution.

SAS9.3 was used for these analyses, and the significance level was 5% on both sides.

## 3 Results

### 3.1 Review of IF reports

We reviewed one meta‐analysis and 14 reports (6 countries) (Boutet et al., 2016; Hartwigsen, Siebner, Deuschl, Jansen, & Ulmer, 2010; Hoggard, Darwent, Capener, Wilkinson, & Griffiths, 2009; Illes, Rosen, et al., 2004; Kaiser et al., 2015; Katzman et al., 1999; Kim et al., 2002; Kumra et al., 2006; Morris et al., 2009; Orme et al., 2010; Reneman et al., 2012; Royal & Peterson, 2008; Sandeman et al., 2013; Seki et al., 2010; Shoemaker et al., 2011). Details of the subject type and IF discovery rate (percentage of individuals in whom IFs were discovered to the total number of participants in whom images were taken) for each of the studies are summarized in Table 2. IF discovery rates were also shown according to their classification by the level of urgency, which has been widely shown in previous studies: Urgency level 1 indicates that immediate referral is required (Immediate referral); urgency level 2 indicates that referral within a few weeks is required (Urgent referral); urgency level 3 indicates that routine referral is required (Routine referral); and urgency level 4 indicates that the finding is common among asymptomatic research participants, and no referral is required (No referral) (Illes, Rosen, et al., 2004; Kaiser et al., 2015; Katzman et al., 1999; Kim et al., 2002; Orme et al., 2010; Reneman et al., 2012; Royal & Peterson, 2008; Sandeman et al., 2013; Seki et al., 2010; Shoemaker et al., 2011) (Table 2).

**Table 2:** Summary of the review of IF discovery rates

Study^a^	Region	Subject (average and range of age)	Number of participants	Overall discovery rate (number)			Urgency level^b^ (% of total participants)			
			(% of males)		Male %	Female%	1	2	3	4
Morris et al. (2009)	Meta‐analysis	16 studies	19,559	0.7 (135)^c^	–		–	–	–	–
		15 studies	15,559	2.0 (375)^d^						
Katzman et al. (1999)	USA	Healthy volunteers (30.6, 3–83)	1000 (54.6)	18.0 (180)	–		0	1.1	1.8	15.1
Kim et al. (2002)	USA	Healthy volunteers (11.2, 0–18)	225 (44.4)	20.9 (47)	29	14.4	0	0.4	8	12.4
Illes, Rosen, et al. (2004)	USA	Healthy volunteers (47.1, 18–90)	151 (54.3)	47.0 (71)	53.7	39.1	0	2	4.6	40.4
Kumra et al. (2006)	USA	Healthy volunteers (–)	60 (–)	13.3 (8)	–		–	–	–	–
Royal & Peterson (2008)	USA	Patients e (–)	397 (60.0)	34.8 (138)	–		0	0.7	2	31.3
		Healthy volunteers (–)	244 (45.1)	27.0 (66)	–		0	0.4	4.7	21.9
Hoggard et al. (2009)	UK	Healthy volunteers (35, 20–81)	525 (62.9)	8.8 (46)	5.2	14.9	–	–	–	–
Hartwigsen et al. (2010)	Germany	Healthy volunteers (25.7, 9–50)	206 (56.8)	19.0 (39)	–		–	–	–	–
Orme et al. (2010)	USA	Research participants (–)^f^	231 (–)^f^	42.9 (99)	–		–	–	–	–
Seki et al. (2010)	Japan	Healthy volunteers (–, 5–8)	110 (53.6)	36.4 (40)	–		0	0.9	1.8	33.6
Shoemaker et al. (2011)	USA	Patients e/ Healthy volunteers	4447 (62.0)	34.1 (1518)	35.2	32.5	0.2	0.7	5.8	34.1
		(30, 0.3–90)								
Reneman et al. (2012)	Netherlands	Healthy volunteers (21.9, 18–35)	201^g^ (45.3)	9.4 (19)	–		0	0.5	3.9	5
		Healthy volunteers (–, 18–35)	180^h^ (–)	30.6 (55)	–		0	0.6	5	25
Sandeman et al. (2013)	UK	Population‐based cohort (72.5, 71–73)	700 (52.6)	31.9 (223)	36.4	26.8	0	0.1	1.3	30.4
Kaiser et al. (2015)	USA	Healthy volunteers (8.3, 0–18)	114 (41.2)	23.2 (26)	15.2^i^	10.6^i^	(1 and 2 combined) 1.8		10.7	10.7
Boutet et al. (2016)	France	Population‐based cohort (75.3, 71–78)	503 (41.4)	77.9 (392)	–	–	–	–	–	–
Range of discovery rates				8.8–77.9			0–0.2	0.1–2.0	1.3–10.7	10.7–40.4

IF, Incidental finding; –, No relevant information was found in the literature.

From the studies of Katzman et al. (1999), Kim et al. (2002), Illes, Rosen, et al. (2004), and Kumra et al. (2006), only the results regarding the findings with potentially significant health impacts are included in the meta‐analysis of Morris et al. (2009).

a Refer to the articles in the reference section.

b Urgency levels 1: immediate referral required (Immediate referral), 2: referral within a few weeks required (Urgent referral), 3: routine referral required (Routine referral), and 4: common findings among asymptomatic research participants and no referral required (No referral) (Katzman et al., 1999).

c Includes only neoplasmic IFs that have the potential to develop symptoms or to impact on preexisting treatments, and that are considered to be clinically significant.

d Includes only nonneoplasmic IFs that have the potential to develop symptoms or to impact preexisting treatments, and that are considered to be clinically significant.

e Patients participated in brain imaging studies (e1: the patient group in the study on neuropsychiatric disorders, e2: the patient group in the study on neuropsychiatric disorders and brain neurology)

f Not known whether the participants were patients or healthy volunteers.

g IFs only in the brain.

h IFs outside the brain (among a total of 201 participants, 180 were evaluated).

i Figures obtained including only findings with urgency levels of 1–3.

The discovery rate of IFs as a whole varied widely, ranging between 8.8 and 77.9%, depending on the report, even after excluding one study that exclusively focused on highly urgent findings (Morris et al., 2009). The reason for this might be attributable to the institutional differences in either the definition of IFs, subject population, or reviewer or judgment process of images. For example, in the reports where the definition of IFs included the changes caused by aging or those considered to be normal variants, the discovery rate was relatively high from 18.0 to 47.0% (Boutet et al., 2016; Illes, Rosen, et al., 2004; Katzman et al., 1999; Kim et al., 2002; Orme et al., 2010; Royal & Peterson, 2008; Seki et al., 2010). In contrast, where IFs were defined as only significant abnormalities (Hoggard et al., 2009), and where normal variants (pineal cysts, hypoplasia of frontal sinus, and others) were excluded from the definition (Reneman et al., 2012), the rate was low, 8.8% and 9.4%, respectively. In addition, the classification of urgency level of findings was not necessarily consistent among studies or even within a study, such that the same types of findings were classified into different urgency levels.

On the other hand, according to a meta‐analysis by Morris et al. (2009), the discovery rates of severe IFs with a significant impact on health and requiring treatment were 0.7% for neoplastic findings and 2.0% for nonneoplastic findings. For all other studies, the discovery rates of IFs of an urgency level of 2 or above were below 2.0% (Illes, Rosen, et al., 2004; Kaiser et al., 2015; Katzman et al., 1999; Kim et al., 2002; Orme et al., 2010; Royal & Peterson, 2008; Seki et al., 2010; Shoemaker et al., 2011). The border between the urgency levels 2 and 3, that is, whether or not the IF requires an urgent examination or treatment, was relatively clear.

In multiple studies, discovery rates of IFs were increased with the age of participants (Boutet et al., 2016; Illes, Rosen, et al., 2004; Morris et al., 2009; Orme et al., 2010; Royal & Peterson, 2008). The number of findings showing white matter lesions and old cerebral infarctions was significantly higher in older participants (Morris et al., 2009). The discovery rate of neoplastic findings increased with age, presumably due to an increase in the prevalence of meningioma (Morris et al., 2009). Some studies reported that the discovery rate itself was higher in older participants; however, findings of a higher level of urgency were more frequently observed in younger participants (including children) (Illes, Rosen, et al., 2004; Royal & Peterson, 2008). In contrast, another study reported that the discovery rates of findings in general, and findings of a high urgency level (urgency level of 2 and above), were higher in participants aged 60 and above (Alphs, Schwartz, Stewart, & Yousem, 2006).

A limited number of studies examined differences in the discovery rate by sex. The discovery rate of IFs as a whole was higher in men in some studies (Illes, Rosen, et al., 2004; Kaiser et al., 2015; Kim et al., 2002; Sandeman et al., 2013; Shoemaker et al., 2011), higher in women in another study (Hoggard et al., 2009), or not different by sex in another study (Orme et al., 2010). Even in studies that reported a higher discovery rate for men, there was no difference by sex in the discovery rate of findings that required further referral (level of urgency 3 and above) (Illes, Rosen, et al., 2004; Kaiser et al., 2015; Kim et al., 2002).

Few studies have compared patient groups and healthy volunteer groups. As a result of meta‐analysis, the discovery rates in patients who participated in research (for cardiovascular diseases, neuropsychiatric disorders, lead exposure (occupational cohort), and others) were significantly higher than those in healthy volunteers (3.4% vs. 1.6%) (Morris et al., 2009). Similarly, one study also reported a higher discovery rate for the patient group (neuropsychiatric disorders including major depressive disorders, oppressive‐compulsive disorder, and attention‐deficit hyperactivity disorder) (Royal & Peterson, 2008).

Some studies reported the results of follow‐up after the detection of IFs, indicating that most IFs could not be identified or did not lead to treatment (Kumra et al., 2006; Orme et al., 2010; Royal & Peterson, 2008; Sandeman et al., 2013). For example, eight participants, with an urgency level of 2 or above underwent a further detailed examination. As a result, the suspected pituitary tumor was not identified in one subject, and in the other seven participants, the findings were confirmed; however treatments were not required (Sandeman et al., 2013). In another study, a subject with an urgency level 2 underwent a follow‐up examination that determined that the tumor or lesion was unlikely to be dangerous (Royal & Peterson, 2008). In yet another study, a subject who was considered to require further referral was followed up for 2 years; however, the finding showed no change and was judged not to be a significant clinical problem (Kumra et al., 2006). Orme et al. performed a follow‐up study to evaluate medical benefits and disadvantages of the detection of IFs for participants, and concluded that among five participants who required further detailed examination, two participants (40%, or 5% of the total IFs) received clear medical benefits (cystic sites, sphenoid sinusitis). In contrast, medical benefit and disadvantage were not clear in three other participants (cysts, signal abnormality, and elevation in nasopharynx) (Orme et al., 2010).

### 3.2 Multi‐institutional study

Table 3 summarizes the number of participants whose brain images were taken within a year of the survey period, the number of participants in whom IFs were discovered, the number by urgency level, and the number of participants informed of IF, for 14 institutions where the survey was conducted. Among the 14 institutions, two were nonmedical organizations/departments (A and L), and IFs were reported at 11 institutions (79%).

**Table 3:** Results of the multi‐institutional study

Institutions^a^	Participants brain imaged	Participants IF discovered (%)	IFs discovered by urgency levels (%)				IFs informed (% of total IFs discovered)	1st survey	2nd survey
			1	2	3	4			
A	743	88 (11.8)	0	0	0	83^b^ (11.2)	0		✓
B	236	10 (4.2)	0	1 (0.4)	0	9 (3.8)	10 (100.0)	✓	✓
C	133	22 (16.5)	0	0	4 (3.0)	18 (13.5)	22 (100.0)	✓	✓
D	128	26 (20.3)	0	0	0	26 (20.3)	0	✓	✓
E	125	25 (20.0)	0	1 (0.8)	18 (14.4)	6 (4.8)	25 (100.0)	✓	✓
F	123	12 (9.8)	0	0	12 (9.8)	0	12 (100.0)	✓	✓
G	98	10 (10.2)	0	0	5^b^ (5.1)	4^b^ (4.1)	1 (10.0)	✓	✓
H	54	29 (53.7)	0	1 (1.9)	28 (51.8)	0	29 (100.0)	✓	✓
I	54	1 (1.9)	0	1 (1.9)	0	0	1 (100.0)	✓	✓
J	38	6 (15.8)	0	0	0	6 (15.8)	2 (33.3)	✓	
K	35	1 (2.9)	0	0	0	1 (2.9)	1 (100.0)	✓	✓
L	76	0	–	–	–	–	–	✓	✓
M	64	0	–	–	–	–	–	✓	✓
N	14	0	–	–	–	–	–	✓	✓
Total	1921	230 (12.0)	0	4 (0.2)	67 (3.5)	153 (8.7)			
Range^c^		0–53.7	0	0–1.9	0–51.8	0–20.3	0–100.0		

a All 14 institutions except A (psychophysiology) and L (economics) were medical research institutions. Three institutions, E, F, and H, conducted studies only in elderly participants.

b Percentages obtained after excluding 6 IFs of unclear urgency (5 at institution A, and 1 at institution G).

c Minimum and maximum discovery rates among those reported by institutions.

#### 3.2.1 Overall discovery rate and details of the findings

Brain images were taken in a total of 1,921 participants at a total of 14 institutions. Among them, IFs were discovered in 230 participants (aged 16–89 years, 41.2 ± 22.0 years, 142 males) (12.0%). The discovery rate differed by institutions, ranging from 0 to 53.7% (average 11.9 ± 14.1%). The most common findings were sinusitis (77 participants), followed by cysts (48 participants), nonspecific white‐matter hyperintensity (26 participants), infarction (22 participants), cavity of the septum pellucidum and cavum vergae (11 participants), atrophy (6 participants), tumor (3 participants), chronic subdural hematoma (two participants), and others.

Findings with the highest urgency level were not discovered at any institutions, but a total of 4 findings with an urgency level of 2 were discovered at four institutions (0.2% of the total participants, 0–1.9% of the participants by institutions). These findings were suspected bone tumor, suspected hydrocephalus, cerebral infarction, and chronic subdural hematoma (Table 4). Among these four participants, two were healthy volunteers, and participation in brain imaging studies provided an opportunity for these findings to be discovered. Findings with an urgency level of 3 were discovered in 3.5% of total participants (67/1921, 0–51.8% by institutions), and findings with an urgency level of four were discovered in 8.7% of total participants (153/1921, 0–20.3% by institutions) (Table 3).

**Table 4:** Summary of 4 participants in whom IFs of an urgency level of 2 were discovered

Subject type	Sex	Age	Disease name	Informing method
Healthy volunteer	F	20	Suspected bone tumor	Verbally explained over the phone after imaging, and referred to the cerebral surgery department of a nearby university hospital
Healthy volunteer	F	46	Suspected hydrocephalus	Verbally informed immediately after the images were taken
Patient	M	65	Cerebral infarction	Verbally informed immediately after the images were taken
Patient	M	81	Chronic subdural hematoma	Verbally informed immediately after the images were taken (visited the cerebral surgery department of the same institution)

#### 3.2.2 Relationship between age and discovery rate

Among the 14 institutions, three institutions conducted studies involving only middle‐aged participants (institutions F, H, and E). For analysis, these three institutions were classified into the elderly research group, and other 11 institutions were classified into the nonelderly research group. The average age of subjects in whom findings were discovered was 68.8 (±11.7) years in the elderly research group, and 29.8 (±13.5) years for the nonelderly research group. The IF discovery rate was significantly higher in the elderly research group at 21.9% (66/302) as compared with 10.1% (164/1619) in the nonelderly research group (chi‐square test, *p *< .0001). In addition, the average discovery rates by institutions tended to be higher in the elderly research group, with an average of 27.8% (±23.0, 9.8–53.7) as compared with an average of 7.5% (±7.1, 0–22.2) in the nonelderly research group (t‐test, *p *= .021).

#### 3.2.3 Relationship between sex and discovery rate

The relationship between sex and discovery rate was analyzed using data from the second survey. Though 13 institutions (all 14 institutions except for institution J) responded to the second survey, institution D did not provide information on the sex of participants. Thus, we used the data from 12 institutions for this analysis. There was no significant difference in the IF discovery rate between male participants (10.9%, 96/884) and female participants (8.7%, 60/691) (chi‐square test, *p *= .151). A similar result was obtained using CMH test (*p *= .149).

#### 3.2.4 Relationship between discovery rate and subject type (patient/healthy volunteer)

Data from 12 institutions after excluding institutions D and J were used for this analysis. No significant difference was found between patients (9.4%, 41/435) and healthy volunteers (10.1%, 115/1140) (chi‐square test, *p *= .694). When data from six institutions (B, C, F, G, I, and M) that took images of both patients and healthy volunteers were analyzed using CMH test, a similar result was obtained (*p *= .776).

#### 3.2.5 Difference in the rate and method of informing participants by institutions

At all institutions, researchers explained to the participants during the IC process that if any findings that needed further examination were incidentally discovered during the course of the evaluation of their brain images taken in the research, researchers will inform them. However, among the institutions that reported discovering IFs, there was a difference in whether or not research participants were informed of the findings (0–100%, Table 3) and in the informing method. Among the 11 institutions that reported discovering IFs, seven institutions (B, C, E, F, H, I, and K), two institutions (G and J), and another two institutions (A and D) informed participants of all of, some of, and none of the findings, respectively. When the informing rate was analyzed using a combination of the type of participants and urgency level (Table 5), participants were informed of all of the findings with an urgency level of 2. In contrast, 92.7% of patients and 96.2% of healthy volunteers were informed of the findings with an urgency level of 3. The informing rate of the findings with an urgency level of 4 was even lower than that of the findings with an urgency level of 3. More than half of the patients (54.3%) compared with only 14.4% of healthy volunteers were informed of the findings with an urgency level of 4. The informing methods included an oral explanation using the console screen immediately after imaging, a written format at a later date, and other methods. Approximately one of five informed patients (11/60) received the information through their physicians.

**Table 5:** Informing rate by subject type and urgency level

	Urgency	Informed		Informing rate (%)
		Yes	No	
Patients^a^	2	2	0	100.0
	3	38	3	92.7
	4	19	16	54.3
Healthy volunteers^b^	2	2	0	100.0
	3	25	1	96.2
	4	17	101	14.4

a One subject whose urgency level was unknown was excluded.

b Five participants whose urgency level and informing status were unknown were excluded.

## 4 Discussion

### 4.1 Tendency of IF rates in previous studies

Over the past decade, several studies on IF discovery have been reported. Our review showed that findings that are considered to have a significant impact on participants’ health are discovered in approximately 2% of asymptomatic research participants, including healthy volunteers. The rate amounted to approximately 20–40% if milder findings were included, with the indication of an especially higher rate in elderly population. These results suggest that all researchers who conduct brain imaging studies should consider implementing an IF handling policy. Above all, in brain imaging studies conducted with 50 or more participants, in studies of dementia, or in population‐based cohort studies in elderly people that involve MRI, measures for managing IFs including how to inform participants of the results have to be discussed, in preparation for the possibility of frequent or serious IF discoveries.

Although a few follow‐up studies have been performed, they showed that more than half of the detected findings required no treatments or were not treatable. (Kumra et al., 2006; Orme et al., 2010; Royal & Peterson, 2008; Sandeman et al., 2013). This reflects that the clinical validity of findings from the brain images is originally vague, which would make it difficult to evaluate the benefit of managing IFs in brain imaging studies. Furthermore, if research participants bear the cost of follow‐up examination, or if the follow‐up period lasts for several years, it is expected that participants assume considerable financial, temporal, and psychological burden. Regarding whether benefits of knowing the presence of findings outweighs such burdens, we would need to know the opinions of people who actually experience such follow‐ups after being informed of an IF through participation in a brain imaging study. Phillips et al. investigated three different stakeholders including research participants who actually received a MRI report from a brain imaging study, and indicated the relationship between participants’ anxiety and the severity of the IFs identified in their brain images (Phillips et al., 2015). Further studies are needed to collect more perspectives of participants and to establish a framework for communicating IFs between researchers and participants.

Moreover, previous studies reached different conclusions regarding the relationship between sex and discovery rate. Regarding subject types, the attributes the participants differed widely in prior studies. Thus, further studies are needed.

### 4.2 Characteristics of overall discovery rate in the multi‐institutional study

The discovery rate of findings with an urgency level of 2 or above, which are considered to have significant health implications, was 1.9% at maximum. Based on our review of previous studies as shown in the first section of Results, we estimated that the discovery rate for findings with an urgency level of 2 or above would be in the range of 0–2%, and the result of the present comparative study also fell within the same range. A study from Japan reported that a brain medical checkup with MRI detected findings that were considered to require further detailed examination in 1.5% of examinees, and findings that do not require further detailed examination but have to be reported to the patient's attending physician were observed in 14.3% (Tsushima, Taketomi‐Takahashi, & Endo, 2005). Since brain medical checkup and research have different imaging purposes and thus use different image resolutions and evaluation processes, a direct comparison of these rates is difficult. However, brain imaging studies should not assume that the number of IFs discovered in the process of research is less than that discovered during medical check‐ups.

The findings with an urgency level of 2 were discovered in four participants, among whom, two were healthy volunteers. All of these participants were immediately informed of the findings. If these research institutions did not have any measures to manage IFs, these findings might have been overlooked. If that had happened, these participants might have had the wrong idea that their brains were free of any health issues. This suggests a possibility of risk of participant's false sense of security about their health (Kirschen et al., 2006), that is a false “clean bill of health” (Royal & Peterson, 2008).

On the other hand, our findings of lower urgency levels of 3 or 4 were discovered at lower rates in general compared with prior studies. At each of the institutions, all images taken were evaluated by physicians in accordance with a standardized policy of the SRPBS. Although a prior study pointed out a concern that radiologists might discover more findings with lower health concerns (urgency level 4) (Royal & Peterson, 2008), the result of this study suggests that this concern might not necessarily be true.

As mentioned above, discovery rate increases with age. However, in our multi‐institutional study, we could not perform a precise comparison, because the age of participants without IFs was unclear. However, studies performed in an older population had a significantly higher discovery rate, which supports the results of previous studies (Boutet et al., 2016; Illes, Rosen, et al., 2004; Morris et al., 2009; Orme et al., 2010; Royal & Peterson, 2008). Regarding the difference in the discovery rate by sex, no difference was observed, whereas there was an inconsistency in previous studies (Hoggard et al., 2009; Illes, Rosen, et al., 2004; Kaiser et al., 2015; Kim et al., 2002; Orme et al., 2010; Sandeman et al., 2013; Shoemaker et al., 2011). In contrast, unlike previous studies (Morris et al., 2009; Royal & Peterson, 2008), no difference was observed in the discovery rate by subject type between healthy volunteers and patients. These results might have been due to the difference in the population between our study and others. All of the patients in this study had psychoneurotic disorders such as depression, autism, and dementia (compared with cardiovascular diseases, neuropsychiatric disorders, lead exposure (occupational cohort), and others in previous studies) (Morris et al., 2009; Royal & Peterson, 2008). We cannot decisively come to a conclusion based only on the results of this study; nonetheless, we can conclude that the possibility of discovering IFs is not low for studies in healthy volunteers, and sufficient measures have to be taken.

Specific epidemiological data should be presented in the explanation document of IC form (Illes et al., 2006; Morris et al., 2009). The IC documents and study protocol for brain imaging studies should include information that findings indicative of a significant health issue might be discovered in 0 to approximately 2% of participants, that the rate might amount to tens of percentages if milder findings are included, and that these findings are more likely to be found in elderly participants, based on the analysis of this study. In addition, it would be better to include specific names of the findings as examples, if needed.

### 4.3 Difference in discovery rate by institutions

As our review showed, IFs discovery rates were different among the previous studies. The difference was also observed in the multi‐institutional study, even when a standardized policy was followed and all images were evaluated by physicians who were capable of interpreting them clinically. We attributed this finding to the following three factors: (1) differences in the definition of IFs; (2) differences in the imaging devices and image types; and (3) differences in the ability of reviewers of images in evaluating the findings. Regarding the difference in the definition of IFs, some institutions (F, H, and I) might have reported only findings with significant health issues as IFs because they reported findings with urgency levels 2 and 3, but not 4. At these institutions, 100% of the findings were informed to the participants, thus these studies may have informed participants of only the IFs that are above a significant threshold. Regarding the two other possibilities, that is, the differences in the imaging devices and capacity of reviewers, we cannot evaluate them based only on the results of this study. Thus, we could not clarify why some institutions (L, M, and N) did not discover any IFs. Perhaps these facilities only regarded the findings with significant health issues as IFs, or maybe these facilities could not detect IFs, including IFs with a low urgency level, due to their insufficient ability to read images.

To adequately protect the participants of a study, at least the findings with an urgency level of 3 or above, which are indicative of health issues, should be judged appropriately. The best approach would be standardization of the capacity of reviewer with specific criteria based on their specialty or years of experience. The existence of a standardized guidance, rather than reliance on each institution's (or research ethics committees’) discretion, would benefit participants. Specific criteria should be ensured by the research ethics committee of each institution. In multi‐institutional collaborative studies where such criteria are applied under various research environments, teleradiology might be useful. Since the initial arrangement of a system would incur a high cost, and be time consuming, the understanding and financial support of funding agencies are essential (Presidential Commission for the Study of Bioethical Issues, 2013). Guidelines for common findings and their urgency levels would also be helpful in reducing the inconsistency among institutions and would mitigate the burden of reviewing physicians. Professional communities, such as an academic society of radiology, could play a role in designing such guidelines. In cases when imaging devices limit clinical judgment, necessity and appropriateness of using such devices should be confirmed through an ethical review, and this situation should be explained with special attention to research participants during the IC process.

### 4.4 Difference among research institutions in the rate of informing participants of findings

As mentioned in the Methods section, considering each institution's respective circumstances, the SRPBS's standardized policy for managing IFs does not include guidance on which findings should be returned to participants or how they should be returned. Consequently, the results of this study indicate a difference in the rate at which participants are informed of IFs, which is the most important issue for research participants’ interests. All IFs with an urgency level 2, which are considered to be indicative of a significant health issue, were verbally explained to participants immediately after they were identified, which is appropriate. However, when we focus on the informing rate of the entire findings, different judgments by institutions emerge. Institutions F, H, and I, which detected findings with an urgency level of 3 only, had an informing rate of 100%. In contrast, institutions A and D, which detected findings with an urgency level of 4 only, did not inform participants of any of the findings. On the other hand, institutions B, C, E, and K informed participants of all of the findings including the ones with an urgency level of 4. Institutions G and J informed participants of some of the findings regardless of the urgency level of 3 or 4. Under the SRPBS, each institution had the discretion to decide whether to inform or not, in consideration of the individuality of the institutions. In other words, participants were informed of their findings by the methods that were devised by individual researchers as approved by institutional review boards. Until our investigation, no study compared the judgment of multiple institutions on whether or not to inform participants of the findings. In this study, we identified these differences, which were considered important for discussing the handling procedures of IFs. This is especially important when inconsistency between IF level and its reporting within an institution exists. It is necessary not only to set a standardized policy, but also to provide education to or promote continuous communication with researchers at institutions.

At the two nonmedical institutions, physicians reviewed all images in accordance with the SRPBS policy, as was performed in medical institutions. However, one institution (A) discovered only findings with an urgency level of 4, about which participants were not informed, whereas the other institution discovered no IFs (L). Some of the medical institutions produced similar results, thus our analysis could not identify issues that are specific to nonmedical institutions. However, such facilities likely experienced difficulties that are specific to nonmedical institutions in establishing an image review system or in providing information or an explanation to participants. Thus, some support system for image review at nonmedical institutions should be developed in the future. For that purpose, surveys targeted to researchers are required.

### 4.5 Necessity of discussion on the informing method

Under the SRPBS, each institution has the discretion of determining whether to inform or not and the informing method. Thus, the multi‐institutional study indicated that each institution used a different informing method. Some institutions reviewed the images just after they were taken and verbally informed participants on the same day. Some institutions reviewed all images together on a later day and informed the participants in writing. All findings with an urgency level of 2 were verbally informed. When patients were involved in a study, there were cases where the patients’ attending physicians informed the patients. Some records were limited to descriptions such as “informed on the day of taking the image” and did not specify who informed the patients. These patients might have been informed through their physician. Thus, the patients’ attending physicians might have had several opportunities to be involved in the informing process.

Generally, in medical research, it is pointed out that the research participants’ level of understanding differs based on the methods used for the explanation in the IC process (Flory & Emanuel, 2004). Similarly, in the process of informing research participants of IFs, the informing methods might affect the participants’ understanding, which can directly lead to a risk of creating anxiety or misunderstanding in participants. Thus, the impact of informing methods on participants’ understanding should be examined in future studies.

Informing a third person of IFs before informing the research participant could be considered an invasion of privacy, even if the person is his/her attending physician. However, for a smooth follow‐up, or when the IF seems to affect the patient's treatment plan, informing the physician of the details of the IFs is considered to be beneficial for the patient. Previous studies indicate that a significant issue is to consider who should be informed of the discovered findings, especially in an environment where there is a family physician system, like in the UK (Booth et al., 2012; Kirschen et al., 2006; Lawrenz & Sobotka, 2008; Wolf, Lawrenz, et al., 2008). These studies conclude that it is best to directly inform the person him/herself, and if this is not possible, prior permission has to be obtained (Illes & Kirschen, 2003; Wolf, Lawrenz, et al., 2008). Although we do not use an official family physician system in Japan, if we inform the patient's attending physician prior to the participant, it should have been clearly explained during the IC process, and consent should have been obtained.

### 4.6 Limitations

Notably, there are four limitations of this study. First, we could not obtain detailed information on the reviewers of images, and could not discuss the effect of different reviewers on the discovery rate and informing rate. In addition, we did not compare situations where physicians are not involved in the evaluation of images. As a result, we could not verify whether our policy was the best. Second, we were not aware of the conditions of investigations at the 39 institutions that did not respond to the SRPBS survey. However, these studies were likely not performed in human participants or did not involve brain imaging technologies, based on the research area of these studies (Takashima et al., 2013). Third, unfortunately, we do not have the complete data on the guidance that institutions provided to participants in the IF report. To focus on the type of information shared regarding an IF and informing method to be used for the research participants, we implemented another study, which included interviews involving the research participants. Fourth, we have not followed up each of the findings discovered in these studies; therefore, we could not demonstrate the possible occurrence of false‐positive or false‐negative findings.

## 5 Conclusion

This present study provides support for the basic data that should be provided in the IC process for future brain imaging studies, including the fact that highly urgent findings might be discovered in approximately 2% of research participants at maximum, including healthy volunteers. Our study is the first study to compare IF discovery rates, informing rates of IFs, and informing methods at multiple institutions. A physician's evaluation of all brain images allowed participants to be immediately informed of their findings with a high urgency level; however, the informing criteria and methods for findings with lower urgency levels (urgency levels of 3 and 4) differ greatly by institution. Notably, informing methods have not been investigated in prior studies. However our study clarified that such methods are critical and potentially determine the impacts of these findings on research participants. We performed another study that included interviews involving research participants who received their IFs information, and our IFs policy will be revised based on the results, with particular focus on the type of IF information to be shared with participants and the method of communication.

## Conflict of Interest

None declared.

## References

[brb3676-bib-0001] Alphs, H. H. , Schwartz, B. S. , Stewart, W. F. , & Yousem, D. M. (2006). Findings on brain MRI from research studies of occupational exposure to known neurotoxicants. AJR. American Journal of Roentgenology, 187, 1043–1047. 16985155 10.2214/AJR.05.0421

[brb3676-bib-0002] Anonymous . (2005). How volunteering for an MRI scan changed my life. Nature, 434, 17. 10.1038/434017a 15744270

[brb3676-bib-0003] Apold, V. S. , & Downie, J. (2011). Bad news about bad news: The disclosure of risks to insurability in research consent processes. Accountability in Research, 18, 31–44. 21287413 10.1080/08989621.2011.542681

[brb3676-bib-0004] Booth, T. C. , Waldman, A. D. , Wardlaw, J. M. , Taylor, S. A. , & Jackson, A. (2012). Management of incidental findings during imaging research in “healthy” volunteers: Current UK practice. The British Journal of Radiology, 85, 11–21. 21937616 10.1259/bjr/73283917 PMC3473920

[brb3676-bib-0005] Borgelt, E. , Anderson, J. A. , & Illes, J. (2013). Managing incidental findings: Lessons from neuroimaging. The American Journal of Bioethics: AJOB, 13, 46–47. 10.1080/15265161.2012.754069 23391062

[brb3676-bib-0006] Boutet, C. , Vassal, F. , Celle, S. , Schneider, F. C. , Barthelemy, J. C. , Laurent, B. , … Roche, F. (2016). Incidental findings on brain magnetic resonance imaging in the elderly: The PROOF study. Brain Imaging and Behavior, 1–7. [Epub ahead of print] 26843003 10.1007/s11682-016-9519-4

[brb3676-bib-0007] Check, E. (2005). Brain‐scan ethics come under the spotlight. Nature, 433, 185. 15662383 10.1038/433185a

[brb3676-bib-0008] Cramer, S. C. , Wu, J. , Hanson, J. A. , Nouri, S. , Karnani, D. , Chuang, T. M. , & Le, V. (2011). A system for addressing incidental findings in neuroimaging research. NeuroImage, 55, 1020–1023. 21224007 10.1016/j.neuroimage.2010.11.091 PMC3057347

[brb3676-bib-0009] Federico, C. , Lombera, S. , & Illes, J. (2011). The Oxford handbook of neuroethics. New York, NY: Oxford University Press.

[brb3676-bib-0010] Flory, J. , & Emanuel, E. (2004). Interventions to improve research participants’ understanding in informed consent for research: A systematic review. JAMA, 292, 1593–1601. 15467062 10.1001/jama.292.13.1593

[brb3676-bib-0011] Fujita, M. , Hayashi, Y. , Tashiro, S. , Takashima, K. , Nakazawa, E. , & Akabayashi, A. (2014). Handling incidental findings in neuroimaging research in Japan: Current state of research facilities and attitudes of investigators and the general population. Health Research Policy and Systems, 12, 58. 25287578 10.1186/1478-4505-12-58 PMC4195876

[brb3676-bib-0012] Grossman, R. I. , & Bernat, J. L. (2004). Incidental research imaging findings ‐ Pandora's costly box. Neurology, 62, 849–850. 15037680 10.1212/01.wnl.0000118214.02495.41

[brb3676-bib-0013] Hartwigsen, G. , Siebner, H. R. , Deuschl, G. , Jansen, O. , & Ulmer, S. (2010). Incidental findings are frequent in young healthy individuals undergoing magnetic resonance imaging in brain research imaging studies: A prospective single‐center study. Journal of Computer Assisted Tomography, 34, 596–600. 20657230 10.1097/RCT.0b013e3181d9c2bb

[brb3676-bib-0014] Hayashi, Y. , Fujita, M. , Takashima, K. , Tashiro, S. , & Akabayashi, A. (2012). Recommendations In Akabayashi A. (Ed.), The management of incidental findings in neuroimaging research (pp. 1–27). The University of Tokyo: Tokyo, In Japanese.

[brb3676-bib-0015] Hilgenberg, S. (2006). Transformation: From medical student to patient. Annals of internal medicine, 144, 779–780. 16702597 10.7326/0003-4819-144-10-200605160-00015

[brb3676-bib-0016] Hoggard, N. , Darwent, G. , Capener, D. , Wilkinson, I. D. , & Griffiths, P. D. (2009). The high incidence and bioethics of findings on magnetic resonance brain imaging of normal volunteers for neuroscience research. Journal of Medical Ethics, 35, 194–199. 19251973 10.1136/jme.2008.025502

[brb3676-bib-0017] Illes, J. (2003). Neuroethics in a new era of neuroimaging. AJNR American Journal of Neuroradiology, 24, 1739–1741. 14561594 PMC7976301

[brb3676-bib-0018] Illes, J. (2006). ‘Pandora's box’ of incidental findings in brain imaging research. Nature Clinical Practice Neurology, 2, 60–61. 10.1038/ncpneuro0119 16932523

[brb3676-bib-0019] Illes, J. , Desmond, J. E. , Huang, L. F. , Raffin, T. A. , & Atlas, S. W. (2002). Ethical and practical considerations in managing incidental findings in functional magnetic resonance imaging. Brain and Cognition, 50, 358–365. 12480483 10.1016/s0278-2626(02)00532-8

[brb3676-bib-0020] Illes, J. , & Kirschen, M. (2003). New prospects and ethical challenges for neuroimaging within and outside the health care system. AJNR American Journal of Neuroradiology, 24, 1932–1934. 14625212 PMC8148911

[brb3676-bib-0021] Illes, J. , Kirschen, M. P. , Edwards, E. , Bandettini, P. , Cho, M. K. , Ford, P. J. , … Seto, B. (2008). Practical approaches to incidental findings in brain imaging research. Neurology, 70, 384–390. 18227420 10.1212/01.wnl.0000280469.17461.94 PMC2605078

[brb3676-bib-0022] Illes, J. , Kirschen, M. P. , Edwards, E. , Stanford, L. R. , Bandettini, P. , Cho, M. K. , … Wolf, S. M. (2006). Incidental Findings in Brain Imaging Research: What should happen when a researcher sees a potential health problem in a brain scan from a research subject? Science, 311, 783–784. 16469905 10.1126/science.1124665 PMC1524853

[brb3676-bib-0023] Illes, J. , Kirschen, M. P. , & Gabrieli, J. D. (2003). From neuroimaging to neuroethics. Nature Neuroscience, 6, 205. 12601375 10.1038/nn0303-205

[brb3676-bib-0024] Illes, J. , Kirschen, M. P. , Karetsky, K. , Kelly, M. , Saha, A. , Desmond, J. E. , … Atlas, S. W. (2004). Discovery and disclosure of incidental findings in neuroimaging research. Journal of Magnetic Resonance Imaging, 20, 743–747. 15503329 10.1002/jmri.20180 PMC1506385

[brb3676-bib-0025] Illes, J. , & Raffin, T. A. (2002). Neuroethics: An emerging new discipline in the study of brain and cognition. Brain and Cognition, 50, 341–344. 12480481 10.1016/s0278-2626(02)00522-5

[brb3676-bib-0026] Illes, J. , Rosen, A. C. , Huang, L. , Goldstein, R. A. , Raffin, T. A. , Swan, G. , & Atlas, S. W. (2004). Ethical consideration of incidental findings on adult brain MRI in research. Neurology, 62, 888–890. 15037687 10.1212/01.wnl.0000118531.90418.89 PMC1506751

[brb3676-bib-0027] Kaiser, D. , Leach, J. , Vannest, J. , Schapiro, M. , Holland, S. , & Cincinnati, M. R. (2015). Imaging of NeuroDevelopment (CMIND) Authorship Consortium. Unanticipated findings in pediatric neuroimaging research: Prevalence of abnormalities and process for reporting and clinical follow‐up. Brain Imaging and Behavior, 9, 32–42. 25403715 10.1007/s11682-014-9327-7

[brb3676-bib-0028] Katzman, G. L. , Dagher, A. P. , & Patronas, N. J. (1999). Incidental findings on brain magnetic resonance imaging from 1000 asymptomatic volunteers. JAMA, 282, 36–39. 10404909 10.1001/jama.282.1.36

[brb3676-bib-0029] Kim, B. S. , Illes, J. , Kaplan, R. T. , Reiss, A. , & Atlas, S. W. (2002). Incidental findings on pediatric MR images of the brain. AJNR American Journal of Neuroradiology, 23, 1674–1677. 12427622 PMC8185842

[brb3676-bib-0030] Kirschen, M. P. , Jaworska, A. , & Illes, J. (2006). Subjects’ expectations in neuroimaging research. Journal of Magnetic Resonance Imaging, 23, 205–209. 16416438 10.1002/jmri.20499 PMC1560341

[brb3676-bib-0031] Kumra, S. , Ashtari, M. , Anderson, B. , Cervellione, K. L. , & Kan, L. (2006). Ethical and practical considerations in the management of incidental findings in pediatric MRI studies. Journal of the American Academy of Child and Adolescent Psychiatry, 45, 1000–1006. 16865043 10.1097/01.chi.0000222786.49477.a8

[brb3676-bib-0032] Lawrenz, F. , & Sobotka, S. (2008). Empirical analysis of current approaches to incidental findings. Journal of Law Medicine & Ethics, 36, 249–255. 10.1111/j.1748-720X.2008.00267.x PMC2578848 18547192

[brb3676-bib-0033] Mamourian, A. (2004). Incidental findings on research functional MR images: Should we look? AJNR American Journal of Neuroradiology, 25, 520–522. 15090335 PMC7975623

[brb3676-bib-0034] Meltzer, L. A. (2006). Undesirable implications of disclosing individual genetic results to research participants. The American Journal of Bioethics: AJOB, 6, 28–30. 10.1080/15265160600935811 17085401

[brb3676-bib-0035] Miller, F. G. , Mello, M. M. , & Joffe, S. (2008). Incidental findings in human subjects research: What do investigators owe research participants? Journal of Law Medicine & Ethics, 36, 271–279. 10.1111/j.1748-720X.2008.00269.x PMC2610459 18547194

[brb3676-bib-0036] Ministry of Education, Culture, Sports, Science and Technology, Japan . Strategic Research Program for Brain Sciences. Available at: http://www.nips.ac.jp/srpbs/. Final accessed on September 22, 2016.

[brb3676-bib-0037] Morris, Z. , Whiteley, W. N. , Longstreth Jr, W. T. , Weber, F. , Lee, Y. C. , Tsushima, Y. , … Al‐Shahi Salman, R. (2009). Incidental findings on brain magnetic resonance imaging: Systematic review and meta‐analysis. BMJ, 339, b3016. 19687093 10.1136/bmj.b3016 PMC2728201

[brb3676-bib-0038] NINDS (National Institute of Neurological Disorders and Stroke) (2005). Detection and disclosure of incidental findings in neuroimaging research. Available at: http://www.ninds.nih.gov/news_and_events/proceedings/ifexecsummary.htm. Final accessed on September 22, 2016.

[brb3676-bib-0039] Orme, N. M. , Fletcher, J. G. , Siddiki, H. A. , Harmsen, W. S. , O'Byrne, M. M. , Port, J. D. , … Wolf, S. M. (2010). Incidental findings in imaging research evaluating incidence, benefit, and burden. Archives of Internal Medicine, 170, 1525–1532. 20876402 10.1001/archinternmed.2010.317 PMC3721142

[brb3676-bib-0040] Parker, L. S. (2008). The future of incidental findings: Should they be viewed as benefits? Journal of Law Medicine & Ethics, 36, 341–351. 10.1111/j.1748-720X.2008.00278.x PMC2586168 18547203

[brb3676-bib-0041] Phillips, J. P. , Cole, C. , Gluck, J. P. , Shoemaker, J. M. , Petree, L. , Helitzer, D. , … Holdsworth, M. (2015). Stakeholder opinions and ethical perspectives support complete disclosure of incidental findings in MRI research. Ethics & Behavior, 25, 332–350. 26877623 10.1080/10508422.2014.938338 PMC4749272

[brb3676-bib-0042] Presidential Commission for the Study of Bioethical Issues (2013). Anticipate and communicate: Ethical management of incidental and secondary findings in the clinical, research, and direct‐to‐consumer contexts. Available at: http://bioethics.gov/sites/default/files/FINALAnticipateCommunicate_PCSBI_0.pdf. Final accessed on September 22, 2016. 10.1093/aje/kwu217 25150271

[brb3676-bib-0043] Reneman, L. , de Win, M. M. , Booij, J. , van den Brink, W. , den Heeten, G. J. , Freling, N. , & Majoie, C. B. (2012). Incidental head and neck findings on MRI in young healthy volunteers: Prevalence and clinical implications. AJNR American journal of neuroradiology, 33, 1971–1974. 22723061 10.3174/ajnr.A3217 PMC7964631

[brb3676-bib-0044] Rosen, B. R. , & Savoy, R. L. (2012). fMRI at 20: Has it changed the world? NeuroImage, 62, 1316–1324. 22433659 10.1016/j.neuroimage.2012.03.004

[brb3676-bib-0045] Royal, J. M. , & Peterson, B. S. (2008). The risks and benefits of searching for incidental findings in MRI research scans. Journal of Law Medicine & Ethics, 36, 305–314. 10.1111/j.1748-720X.2008.00274.x PMC4290840 18547199

[brb3676-bib-0046] Sandeman, E. M. , Hernandez Mdel, C. , Morris, Z. , Bastin, M. E. , Murray, C. , Gow, A. J. , … Wardlaw, J. M. (2013). Incidental findings on brain MR imaging in older community‐dwelling subjects are common but serious medical consequences are rare: A cohort study. PLoS ONE, 8, e71467. 23967214 10.1371/journal.pone.0071467 PMC3744549

[brb3676-bib-0047] Seki, A. , Uchiyama, H. , Fukushi, T. , Sakura, O. , & Tatsuya, K. (2010). Japan Children's Study Group. Incidental findings of brain magnetic resonance imaging study in a pediatric cohort in Japan and recommendation for a model management protocol. Journal of Epidemiology, 20(Suppl 2), S498–S504. 20179362 10.2188/jea.JE20090196 PMC3920402

[brb3676-bib-0048] Shaw, R. L. , Senior, C. , Peel, E. , Cooke, R. , & Donnelly, L. S. (2008). Ethical issues in neuroimaging health research: An IPA study with research participants. Journal of Health Psychology, 13, 1051–1059. 18987078 10.1177/1359105308097970

[brb3676-bib-0049] Shoemaker, J. M. , Cole, C. , Petree, L. E. , Helitzer, D. L. , Holdsworth, M. T. , Gluck, J. P. , & Phillips, J. P. (2016). Evolution of universal review and disclosure of MRI reports to research participants. Brain and Behavior, 6, e00428. 26893955 10.1002/brb3.428 PMC4744862

[brb3676-bib-0050] Shoemaker, J. M. , Holdsworth, M. T. , Aine, C. , Calhoun, V. D. , de La Garza, R. , Ewing, S. W. F. , … Phillips, J. P. (2011). A practical approach to incidental findings in neuroimaging research. Neurology, 77, 2123–2127. 22131543 10.1212/WNL.0b013e31823d7687 PMC3235350

[brb3676-bib-0051] Takashima, K. , Tashiro, S. , Tsuchiya, A. , Fujita, M. , & Takimoto, Y. (2013). A practical approach to handle incidental findings in brain imaging studies: A survey appraisal of introducing a standardized method for the management of incidental findings to the organizations participating in the Strategic Research Program for Brain Sciences In Akabayashi A. (Ed.). Trends in neuroethics 2012 (pp. 5–42). The University of Tokyo: Tokyo, In Japanese.

[brb3676-bib-0052] Tsushima, Y. , Taketomi‐Takahashi, A. , & Endo, K. (2005). Prevalence of abnormal findings on brain magnetic resonance (MR) examinations in adult participants of brain docking. BMC Neurology, 5, 18. 16207376 10.1186/1471-2377-5-18 PMC1266373

[brb3676-bib-0053] Underwood, E. (2012). Neuroethics. When a brain scan bears bad news. Science, 338, 455. 23112304 10.1126/science.338.6106.455

[brb3676-bib-0054] Wardlaw, J. M. , Davies, H. , Booth, T. C. , Laurie, G. , Compston, A. , Freeman, C. , … Jackson, A. (2015). Acting on incidental findings in research imaging. BMJ, 351, h5190. 26556813 10.1136/bmj.h5190

[brb3676-bib-0055] Warlow, C. (2011). Neuroimaging–chance effects and unintended consequences. Cortex, 47, 1270–1271. 21621763 10.1016/j.cortex.2011.04.015

[brb3676-bib-0056] Wolf, S. M. (2008). Introduction: The challenge of incidental findings. Journal of Law Medicine & Ethics, 36, 216–218. 10.1111/j.1748-720X.2008.00265.x PMC2587006 18547190

[brb3676-bib-0057] Wolf, S. M. , Lawrenz, F. P. , Nelson, C. A. , Kahn, J. P. , Cho, M. K. , Clayton, E. W. , … Wilfond, B. S. (2008). Managing incidental findings in human subjects research: Analysis and recommendations. Journal of Law Medicine & Ethics, 36, 219–248. 10.1111/j.1748-720X.2008.00266.x PMC2575242 18547191

[brb3676-bib-0058] Wolf, S. M. , Paradise, J. , & Caga‐anan, C. (2008). The law of incidental findings in human subjects research: Establishing researchers’ duties. Journal of Law Medicine & Ethics, 36, 361–383. 10.1111/j.1748-720X.2008.00281.x PMC2581517 18547206

